# Characteristics of Traumatic Urogenital Injuries in Emergency Department; a 10-year Cross-sectional Study 

**Published:** 2019-11-09

**Authors:** Babak Javanmard, Morteza Fallah-karkan, Mohammadreza Razzaghi, Anahita Ansari Djafari, Saleh Ghiasy, Behzad Lotfi, Reza Vafaee

**Affiliations:** 1 Urology Department, Shohada e Tajrish Hospital, Shahid Beheshti University of Medical Sciences, Tehran, Iran.; 2 Laser Application in Medical Science Research Center, Shahid Beheshti University of Medical Sciences, Tehran, Iran.; 3 Proteomics Research Center, Shahid Beheshti University of Medical Sciences, Tehran, Iran.

**Keywords:** Urogenital system, acute kidney injury, wounds and injuries, multiple trauma, epidemiology, mortality

## Abstract

**Introduction:**

Urogenital system injuries (UGIs) are seen in 10% of adult cases with multiple trauma. Although UGIs are rarely life threatening, they can cause major long-term morbidities. This study aimed to evaluate the characteristics of traumatic UGIs in patients who were referred to emergency department following multiple traumas.

**Methods:**

This retrospective cross-sectional study was conducted on multiple trauma patients who were presented to emergency department during a 10-year period (2008-2017). All patients with kidney, ureter, bladder, urethra, or external genitalia injuries were studied. The patients’ data were extracted from their clinical profiles.

**Results:**

Out of the 13598 admitted patients in our trauma center, UGIs were seen in 267 (1.9%) cases. The mean age of patients with UGIs was 27.3 ± 6.1 years (74.15% male). The highest incidence of UGI was seen in those aged between 21 and 30 years (39.7%) and motorcycle accidents (49%) was the most frequent cause of trauma. 221 patients had an unstable situation and were emergently transferred to operation room (13.57% with traumatic kidney injury). The most common injured sites of urogenital system were kidney with 155 (58%) cases, followed by external genitalia with 91 (34.1%) cases. 77.5% of cases were managed conservatively and the rest (22.5%) underwent surgical procedures.

**Conclusion:**

UGIs comprise a low percentage (2%) of traumatic injuries, which are mostly caused by blunt trauma due to road traffic accidents. Kidney is the most common injured organ and UGIs mostly happen in young ages.

## Introduction

With economic development, incidence of traffic and industry-related accidents, iatrogenic injury, intoxication and also psychological self-mutilation have been increasing (1-8). Morbidity due to trauma has been one of the most serious public health concerns all over the world (9).

In most reports, urogenital system injuries (UGIs) are seen in approximately 10% of adult traumatic patients and in less than 3% of children with multiple/severe trauma of lower abdomen or pelvis (10-12). Kidney is the most common injured organ in genitourinary system while ureteral traumas are rare (1, 13). Although UGIs are rarely life threatening, they can cause significant long-term morbidities such as renal function impairment, sexual dysfunction and urethral stricture (14-16).

Finding an ideal management in patients with UGIs requires comprehensive epidemiological information in different populations and times (14, 17). Therefore, this study aimed to evaluate the characteristics of traumatic UGIs in patients who were referred to emergency department following multiple traumas. 

## Methods

***Study design and setting***

This retrospective cross-sectional study was conducted on multiple trauma patients who were presented to emergency department of Shohada-e-Tajrish Hospital, Tehran, Iran, during a 10-year period (2008-2017). This hospital is the main referral trauma center in north and northeast of Tehran. The Ethics committee of Shohada-e-Tajrish Hospital approved this study and gave us permission to review patients’ medical data. Considering the retrospective design of the study, the ethical committee ceded the necessity to have patients provide consent for collecting their medical data. The patients’ data were collected confidentially and de-identified.

***Participants***

All admitted patients for more than 6 hours with any kind of traumatic UGIs such as kidney, ureter, bladder, urethra, external genitalia and adrenal gland injuries, were included using a census sampling method. Patients with incomplete medical profiles or unclear evidence regarding the urogenital tract were excluded. There was not any age or gender limitation.

***Data gathering***

We reviewed all information regarding demographic characteristics (age, gender), history, physical examination, mechanism of injury, type and severity of UGIs, associated injuries, done procedures, and also type of management (conservative or surgical managements) by referring to the medical records of the cases and filling out a checklist designed for the investigation. The type and mechanism of the injury were adapted according to the International Classification of Diseases, 10th revision (ICD-10)(18). Two senior urology residents (MFk and SG) were responsible for data gathering.

***Statistical Analysis***

Statistical analyses were performed by the Statistical Package of Social Science System software, version 20. Findings were presented using descriptive statistics as mean ± standard deviation or frequency (%). 

## Results

***Baseline characteristics of studied patients***

Out of 13598 admitted patients in our trauma center during the study period, UGIs were seen in 267 (1.9%) patients (92.5% blunt and 7.5% penetrating trauma). Table 1 shows the baseline characteristics of studied patients. The mean age of patients with UGIs was 27.3 ± 6.1 (3 - 82) years (74.15% male). The highest incidence of UGI was seen in those aged between 21 and 30 years (39.7%) followed by 11-20 years (25.07%) and 3 -10 years (19.1%) age groups. Motorcycle accidents (49%) followed by falling down (26%) were the most frequent causes of trauma. 221 patients had unstable situation and were emergently transferred to operation room (13.57% with traumatic kidney injury). 7 (2.8%) cases died due to severity of associated injuries (5 patients had renal and 2 cases had bladder injuries). The most common injured sites of urogenital system were kidney with 155 (58%) cases, followed by external genitalia with 91 (34.1%) cases (table 2). The common associated injuries were extremity (52.8%) and abdominopelvic (41.9%) injuries.

***Management strategies***

Table 3 summarizes different types of treatment strategies used for management of injured patients. 

*Upper urinary tract*

Conservative management was applied for 51.8% of patients with renal injury and 2 patients with grade IV renal injury underwent angiographic embolization. Two patients with adrenal trauma were managed conservatively. 

*Lower urinary tract*

Nineteen cases out of 27 patients with extra peritoneal bladder rupture were managed conservatively, by foley catheter insertion for two weeks. In one case with extra peritoneal bladder rupture, during orthopedic surgery bladder was injured intra peritoneally so cystoraphy was done intraoperatively. Fourteen of 25 patients with testis trauma had intact tunica albuginea and underwent conservative management. Three patients needed dermal flap to reform scrota because of extensive external genitalia injuries. Nine men and one girl with external genitalia injuries were managed conservatively. Ten of fifteen patients with urethral injuries underwent primary realignment (seven cases had complete urethral injuries). For five patients cystostomy was performed and they became candidates for delayed treatment.

**Table 1:** Baseline characteristics of studied patients

**Variables **	**Number (%)**
**Gender**	
Male	198 (74.15)
Female	69 (25.8)
**Age (year)**	
3 – 10	51 (19.1)
11 -20	67 (25.07)
21 -30	106 (39.7)
30 – 60	31 (11.4)
>60	12 (4.73)
**Trauma mechanism**	
Motorcycle accident	131(49)
Falling down	69 (26)
Car accident	58 (21.6)
Sports accident	9 (3.3)
**Hemodynamics **	
Stable	238 (89.2)
Unstable	29 (10.8)
**Associated injuries**	
Abdominopelvic	104 (38.8 )
Extremities	85 (31.9)
Thoracic	46 (17.1)
Head and neck	32(12.2)
**Presenting sign and symptom**	
Hematuria	169 (63.2)
Flank pain	50 (19.1)
Flank or genital abrasion	19 (7.1)
Obstruction	15 (5.6)
Other	14 (5.2)

**Table 2:** Distribution of urogenital injuries among the studied subjects

**Injured organ**	**Number (%)**
**Upper urinary tract**	
Renal	155 (58.1)
Ureter	3 (1.1)
Adrenal	2 (0.7)
**Lower urinary tract **	
Scrotum	43 (16.1)
Testis	25(9.3)
Urethra	15(5.6)
Bladder	34 (12.7)
Genitalia	25 (9.9)
**Severity of renal injury**	
Grade 1	103 (38.5)
Grade 2	34 (12.7)
Grade 3	10 (3.7)
Grade 4	5 (1.8)
Grade 5	3 (1.1)
**Type of bladder injury **	
Intra peritoneal injury	7 (2.6)
Extra peritoneal injury	27 (10.1)
**Type of ureteral injuries**	
Partial rupture of urethra	10(3.7)
Complete rupture of urethra	5(1.8)

**Table 3:** Management strategies of studied patients

**Variables**	**Number (%)**
**Type of management**	
Conservative management	207(77.5)
Operative management	52(19.4)
**Type of procedures**	
Nephrectomy	7(2.6)
Bladder repair	8(2.9)
Immediate endoscopic realignment	10(3.7)
Renoraphy	10(3.7)
Ureter repair	2(0.7)
Partial orchiectomy	5(1.8)
Male genital organ repair	8(2.9)
Female genital organ repair	2(0.7)

## Discussion

According to our results, the frequency of UGIs in this series of cases was 1.9%. The most common injured sites of urogenital system were kidney with 155 (58%) cases, followed by external genitalia with 91 (34.1%) cases. 77.5% of cases were managed conservatively.

Appropriate data are needed to assess clinical presentation, therapeutic procedure, trauma systems issues, and quality of care for this health problem (19). According to burden of disease and injuries, 28% of years of life lost in Iran is attributed to accidents (20). This issue may point out that Emergency Medical Service is not able to provide trauma patients with efficient and perfect pre-hospital care (21).

The condition of trauma possibly depends on the space from hospital to high ways, socioeconomic status, grade of hospital as trauma center, and public health infrastructure mainly in prehospital trauma care (22, 23). Abundance of construction projects may be the reason that falling down is the second most common cause of injuries.

According to the obtained results from this study, UGIs comprise a low proportion of injuries in trauma patients (267 of 24742 patients, about 2 %). This is similar to the findings of other studies (10, 14, 24-26).

Presentations of injured patients were similar to those in the literature. Regarding gender, the males enrolled were four times the females, and mainly prevalent in the 3rd decade of life (14, 17, 27-29). This may owe to men working in more risky jobs, being more involved in violent behavior and also number of male drivers being more than females (30). Most traffic accidents involved middle-aged victims, while the pedestrian accidents involved older age patients.

Road traffic accidents are the cause of the majority of UGIs; trauma had a blunt nature in 92.5% of patients, which is almost similar to other studies (13, 31-34). Moreover, kidney was the most common injured organ and kidney repair was the most common surgical treatment in this study. The haemodynamic condition is the point of reference for the diagnostic and therapeutic approach in trauma patients. Haemodynamically unstable patients need urgent laparotomy (35).

Initial treatment of patients with posterior urethral injuries (PUI) is one of the most challenging and technically difficult problems in urology (36). The controversy in treatment of PUI is surrounding two basically different approaches; one is the insertion of suprapubic cystostomy followed by a delayed urethroplasty; and the other approach is concomitant one stage suprapubic cystostomy and urethral realignment (37, 38). Advances in endoscopic techniques have facilitated early realignment and transurethral catheterization as a new treatment approach. In the present study, two thirds of the urethral injury cases underwent primary realignment and the rest became delayed urethroplasty candidates after cystostomy.

Skin and tissue defects are another problem in patients with trauma. Grafts can be utilized for extended skin and tissue defects (39, 40). Complete avulsion of the scrotum and penis skin, creates a very serious life-threatening problem. Also, there is a serious problem in saving the testes, covering the penis and rehabilitation of area function.

There are two types of bladder ruptures: intraperitoneal and extraperitoneal. The extraperitoneal ruptures are often associated with pelvic fractures. Intraperitoneal ruptures often happen as a result of frontal impact, with unexpected deceleration while having a full bladder (24). The two most common symptoms of bladder injuries are macroscopic hematuria and abdominal tenderness. Other findings consist of inability to void, bruising of the supra-pubic region, and contrast extravasations in computed tomography (CT) urography. Pelvic fractures, which are associated with multiple organ injures, including bladder, have a considerable mortality rate (20–40 %) (11, 41). According to our findings, due to the low rate of positive imaging findings in stable trauma patients with microscopic hematuria, it seems that there is no need to routinely perform abdominopelvic CT scan with intravenous contrast in these patients. 

Since most cases with urogenital trauma have multiple injuries, they need a multidisciplinary approach; disregarding these injuries may cause severe complications. We propose establishing an integrated trauma system in Iran to improve the quality of trauma care. Also, preventive programs aimed at behavioral modifications in the society should be performed to decrease the health and economic burdens imposed by accidents. 

## Limitation

The first limitation of this investigation is the retrospective nature of the study, which caused some missing medical history and physical examination data. The second limitation is the lack of follow up assessments of disease adjusted life years. 

## Conclusion

UGIs mostly result from blunt trauma due to road traffic accidents. Renal injuries are the most common; and UGI mostly develops in youth. Conservative management can be the treatment of choice for renal trauma patients who are hemodynamically stable.
